# Chitosan as Functional Biomaterial for Designing Delivery Systems in Cardiac Therapies

**DOI:** 10.3390/gels7040253

**Published:** 2021-12-09

**Authors:** Bhaumik Patel, Ravi Manne, Devang B. Patel, Shashank Gorityala, Arunkumar Palaniappan, Mallesh Kurakula

**Affiliations:** 1 Product Development, Cure Pharmaceutical Corporation, Los Angeles, CA 90025, USA; bhaumikp17@gmail.com; 2 Chemtex Environmental Laboratory, Quality Control, and Assurance Department, Port Arthur, TX 77642, USA; ravimannemr@gmail.com; 3 Department of Pharmaceutical Sciences, Arnold and Marie Schwartz College of Pharmacy and Health Sciences, Long Island University, Brooklyn, NY 11201, USA; devang513@gmail.com; 4 Department of Bioanalytical Chemistry, Covance Laboratories, Madison, WI 53704, USA; Shashank.gorityala@covance.com; 5 Centre for Biomaterials, Cellular, and Molecular Theranostics, Vellore Institute of Technology, Vellore 632014, India; arunkumarp@vit.ac.in

**Keywords:** chitosan, drug delivery systems, myocardial infarction, cardiac therapies

## Abstract

Cardiovascular diseases are a leading cause of mortality across the globe, and transplant surgeries are not always successful since it is not always possible to replace most of the damaged heart tissues, for example in myocardial infarction. Chitosan, a natural polysaccharide, is an important biomaterial for many biomedical and pharmaceutical industries. Based on the origin, degree of deacetylation, structure, and biological functions, chitosan has emerged for vital tissue engineering applications. Recent studies reported that chitosan coupled with innovative technologies helped to load or deliver drugs or stem cells to repair the damaged heart tissue not just in a myocardial infarction but even in other cardiac therapies. Herein, we outlined the latest advances in cardiac tissue engineering mediated by chitosan overcoming the barriers in cardiac diseases. We reviewed in vitro and in vivo data reported dealing with drug delivery systems, scaffolds, or carriers fabricated using chitosan for stem cell therapy essential in cardiac tissue engineering. This comprehensive review also summarizes the properties of chitosan as a biomaterial substrate having sufficient mechanical stability that can stimulate the native collagen fibril structure for differentiating pluripotent stem cells and mesenchymal stem cells into cardiomyocytes for cardiac tissue engineering.

## 1. Introduction

**Figure 1:**
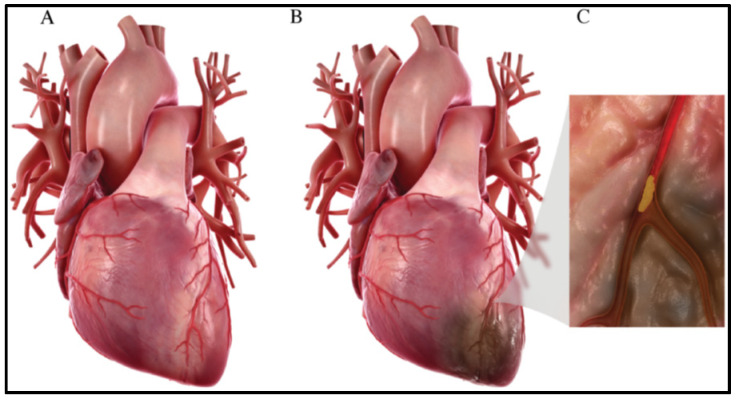
(**A**) Normal heart. (**B**) Heart after myocardial infarction, where an ischemic region is developed due to no/reduced blood flow to that cardiac region. (**C**) Plaque accumulation in the coronary artery that leads to its blockage [3].

Cardiovascular diseases (CVD) are one of the leading causes of death worldwide. According to a 2015 report, CVD accounted for 17.92 million deaths per year [1]. CVD are diseases that relate to the blood vessels and heart, which include a wide gamut of diseases: coronary artery/heart diseases (CAD/CHD) such as angina and myocardial infarction, stroke, heart failure, hypertensive heart disease, rheumatic heart disease, cardiomyopathy, abnormal heart rhythms, congenital heart disease, valvular heart disease, carditis, aortic aneurysms, peripheral artery disease, thromboembolic disease, venous thrombosis, etc. [1]. CHD is one of the most common CVD, which affects 117.79 million people across the globe and accounted for 8.92 million deaths in the year 2015 [1]. CHD occurs when there is a thinning or blockage of the coronary arteries, which supply blood to the heart (a condition called atherosclerosis) [2], as shown in Figure 1. This happens due to the accumulation of plaque/thrombus on the inner linings of the arteries. This results in the limited or disrupted supply of oxygen and nutrients to the heart, resulting in ischemic condition and leading to the death of cardiac tissue (more specifically, there occurs a loss of about one billion cardiomyocytes). This condition is called a myocardial infarction (MI) or heart attack. This leads to the adverse remodeling of the both left and right ventricular regions of the heart and finally leads to heart failure in extreme cases [2].

## 2. Conventional Treatment Modalities

The conventional treatment modalities include the administration of pharmaceutical drugs (anticoagulants, platelet inhibitors, cholesteryl ester transfer protein (CETP) inhibitors, and β-blockers), surgical interventions, mainly implantation of a pacemaker, metallic/biodegradable drug-eluting stents, implantable cardioverter–defibrillator (ICD), coronary artery bypass graft surgery (CABG), and heart transplantation in extreme heart failure cases [4,5,6,7]. The main drawbacks of the conventional treatment modalities (pharmaceutical drugs administration and surgical interventions) are that they focus only on the opening of the blocked arteries as well as on the mitigation of the symptoms without addressing the regeneration of lost cardiac tissue in the ischemic region. On the other hand, the limited availability of donors and immune rejections are important barriers in the case of heart transplantation [8]. Although there are multiple immunosuppressants in use to avoid immune rejections, there is a need for more data to determine which patient receives which type of immunosuppressant.

Another approach for this is complementary and alternative medicine (CAM), which plays a significant role in the treatment of cardiovascular disease. CAM is generally defined as a group of diverse medical and health care systems, practices, and products that are not generally considered as part of conventional medicine. Some of the CAM-based approaches are biologically-based therapies, mind–body therapies, manipulative and body-based therapies, whole medical systems, and energy medicine [9].

## 3. Cardiac Tissue Engineering

Currently, researchers across the globe are working toward the regeneration of damaged cardiac tissues through various means. Some of the techniques for the regeneration of the lost cardiac tissues include the administration of appropriate cells of interest, biomaterials, growth factors, and immune-modulatory factors or the combination of either a few or all of the above [8]. The administration of appropriate cells of interest for the repair of cardiac tissue, also known as cellular cardiomyopathy, is one of the most widely researched areas in the cardiac regeneration field. In this, the progenitor cells or the stem cells are injected into the infarct heart region, which could replace the dead cardiomyocytes [9,10,11,12]. Some of the cells typically used are bone marrow stem cells (BMCs), mesenchymal stem cells (MSCs), embryonic stem cells (ESCs), hematopoietic stem cells (HSCs), induced pluripotent stem cells (iPSCs), and cardiac progenitor cells (CPC) [10,11,12,13]. The potential of these cells to improve functions in heart failure cases is reported through a few animal models studies as well as in a few human clinical trials as well. However, few other studies prove that there is no significant effect of these cell therapies [14,15,16].

Among these, biomaterials form the most important basic component through which other regenerative factors such as cells, growth factors, or other immune-modulatory factors could be delivered in the ischemic region. The advantages of using biomaterials for the delivery of cells are that they act as an extracellular matrix (ECM)-like medium, which provides the required binding sites for the cells as well as help in the retention of the cells in the desired region longer when compared to administration of cells alone. In addition, in the case of the delivery of growth factors and immune-modulatory factors, these biomaterials provide a platform for the controlled or sustained release of these factors. Moreover, these materials give stability as well as protect these compounds from the rapid biodegradation inside the body [17,18,19,20,21]. In the case of cardiac regeneration, polymeric biomaterials are a widely preferred choice as they have mechanical properties similar to that of the cardiac tissues. Some of the most widely used forms in which these biomaterials are used include hydrogels, nanofibrous cardiac patch/scaffolds, microspheres, nanoparticles, or a combination of one or two of them [20,21].

## 4. Role of Chitosan in Tissue Engineering

Chitosan is derived from chitin by the deacetylation of the chitin. Chitosan is one of the few polymers that is similar to glycosaminoglycans (GAG) that are widely distributed throughout the connective tissues, which makes it an ideal choice for tissue engineering applications [22]. In addition, the existence of the free amino groups in its backbone chain enables further chemical modifications for biomedical functionality. The current cardiac tissue engineering research aims at designing the tissue constructs to support, replace, repair, enhance, as well as restore the functionality of the injured or diseased myocardial tissue. The initial focus to achieve cardiac tissue engineering was to directly inject the viable cells into the infarcted myocardium tissue; however, this strategy suffers from limited cell retention and poor cell survival. An alternative and promising strategy to overcome these limitations is the incorporation of the biomaterial within the heart wall in direct contact with the cardiac cells. In this approach, the natural or synthetic materials are injected in a combination of various biomaterials or cells. Chitosan is a natural polymer obtained from the shell of shellfish and is considered one of the most abundant organic materials. It is made up of a polymer composed of glucosamine and N-acetyl glucosamine units linked by β (1–4) glycosidic bonds, and the characteristics of biocompatibility, biodegradability, antibacterial, as well as wound healing make it an ideal biomaterial used for tissue engineering activities. The antibacterial activities of chitosan have been explored widely and reported [23]. These characteristics of chitosan have been shown to enhance cell engraftment and survival, contributing to myocardial repair.

## 5. Chitosan Scaffolds in Building Functional Cardiac Tissue

Injectable scaffolds are a promising therapeutic approach for cardiac tissue regeneration in case of progressive heart failure following myocardial infarction. Chitosan is mucoadhesive, hemostatic, and capable of binding with cell membranes due to the presence of the positively charged amino acid groups. Chitosan also has the ability to form scaffolds that are well interconnected with adequate porosity to support cell viability by a consistent supply of oxygen and nutrients [24]. Another key feature of a chitosan-based scaffold is the controlled delivery of the loaded therapeutic molecules and growth factors. This makes them an ideal candidate for tissue engineering and cardiac tissue regeneration. Chitosan is a biocompatible substrate that acts as an extracellular matrix where the immobilized angiogenic growth factors induce the cellular responses that could stimulate the migration and proliferation of endothelial cells to ultimately facilitate the formation of the new vascularized network [25]. Studies have shown that the porcine ECM is cross-linked with chitosan and genipin. This facilitates the preservation of ECM biological composition and also increases the mechanical strength of the injectable scaffolds. The decellularization of non-clinical ECM was used to reduce the immunogenicity impact before using it as a scaffold for tissue engineering.

**Figure 2:**
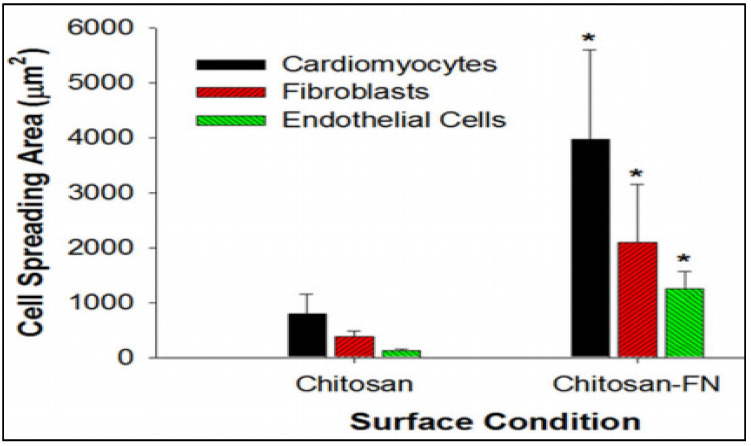
The comparison of the cardiomyocytes, fibroblasts, and endothelial cells spreading area on chitosan nanofibers immobilized with and without fibronectin. Immobilization of fibronectin enhanced the cellular spreading resembling the native heart tissue. Data are expressed as means SD. * *p* < 0.01 (chitosan vs. chitosan-FN). [26].

Another promising strategy in cardiac tissue regeneration is the in vitro generation of the three-dimensional (3D) myocardial tissue-like construct employing cells, biomaterials, and biomolecules. The challenge with this approach is to maintain the functional characteristics of the cardiac myocytes for a long-term culture and treatment period. Researchers have been successful in the fabrication of bioactive 3D chitosan nanofiber scaffolds using the electrospinning technique and evaluating the long-term cardiac function in the 3D co-culture model [26]. The cellular attachment to the chitosan nanofibers and the infiltration into the interfibrous spaces were found to be enhanced by the immobilization of fibronectin onto the chitosan nanofibers by adsorption. Figure 2 represents the comparison of the cardiomyocytes, fibroblasts, and endothelial cell spreading area in the presence and absence of fibronectin. The cells cultured on the fibronectin-coated chitosan films demonstrated enhanced cellular spreading, a significant increase in vinculin expression, and enhanced fibrous F-actin cytoskeleton [26].

CHD is the major cause of death worldwide and is mainly caused by the accumulation of the circulating cholesterol on the artery walls, narrowing arteries and leading to reduced blood flow to the heart. Among the commonly used polysaccharides, chitosan oligosaccharide (COS), which is the degradation product of chitosan via chemical hydrolysis or enzymatic degradation involving a deacetylation and depolymerization process, has shown some promising remedies for CHD. COS is an effective antiatherosclerosis agent [27]. Studies have shown that the consumption of COS has increased the values of the left ventricular ejection fraction (LVEF) compared to the control group, which did not consume COS. LVEF is an important predictor of heart failure-related hospitalization and mortality in ambulatory adults with CHD [28]. However, the molecular mechanism between the COS and improved condition in CHD patients is not fully understood.

## 6. Chitosan in Conjugation with Other Polymers and Its Use in Cardiac Therapies

**Figure 3:**
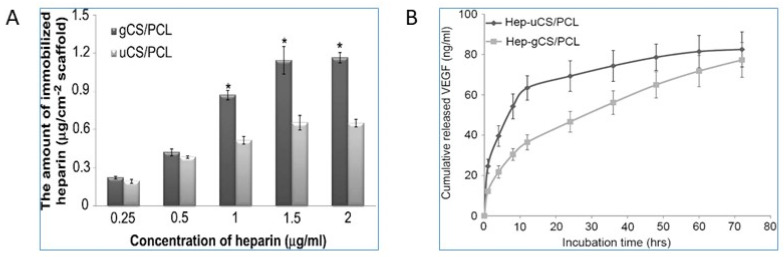
Comparison of the gradient and uniform CS/PCL in the cardiac tissue regeneration [29]. (**A**) Heparinization of the uniform CS/PCL (uCS/PCL) and gradient (gCS/PCL); (**B**) Cumulative release of VEGF from heparinized-uniform CS/PCL (Hep-uCS/PCL) and heparinized-gradient CS/PCL (Hep-gCS/PCL). Values represent mean ±SD (*n* = 5). “*” indicate statistically significant differences compared to heparinized groups (*p* < 0.05).

The major limitation for the tissue-engineered small-diameter blood vessels is restenosis, where the part of the artery that was treated for blockade becomes narrow again due to thrombopoiesis. Studies have shown that the graded chitosan/ε-caprolactone (CS/PCL) nanofibrous vessel scaffolds were immobilized with the vascular endothelial growth factor (VEGF) as an approach to creating small-diameter blood vessel grafts with innate properties of mammalian vessels of anticoagulation and rapid induction of re-endothelialization [29]. Heparin biomaterials are typically used for the immobilization and sustained release of VEGF. It is reported that the amount of conjugated heparin on gradient CS/PCL was twice as high as the uniform CS/PCL, which helped in the enhanced sustained release of VEGF that has shown the rapid induction of endothelialization for cardiac tissue regeneration, as shown in Figure 3.

**Figure 4:**
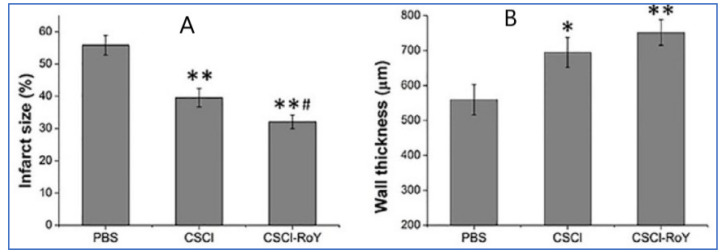
Impact of CSCL-RoY thermoresponsive hydrogel on myocardial infarction [31]. (**A**) Quantitative analysis of infarct size after the injections; (**B**) Quantitative analysis of infarct wall thickness (* *p* < 0.05 vs. PBS group, ** *p* < 0.01 vs. PBS group, # *p* < 0.05 vs. CSCl group).

Chitosan-based hydrogels are known to respond to a variety of external stimuli such as temperature and light and assemble as interconnected porous structures to help in cell infiltration. Thermoresponsive chitosan hydrogels are the popular choice, as the cells are easy to incorporate in the polymer solution [30]. Once the hydrogel is exposed to the body temperatures, the polymeric solution becomes the gel in a short period, localizing the cells within the injected area. In addition, researchers have developed the thermosensitive chitosan chloride-RoY (CSCL-RoY) hydrogel to improve angiogenesis under hypoxia in myocardial infarction patients, which is a major challenge in cardiac repair. The data suggest that the infarct size significantly decreased after the injection of CSCL-RoY hydrogel compared to the injection of PBS or CSCL hydrogel (Figure 4A). A similar positive impact was observed in the ventricular wall thickness in the center of the infarct zone after the injection of CSCL-RoY hydrogel (Figure 4B) [31].

**Figure 5:**
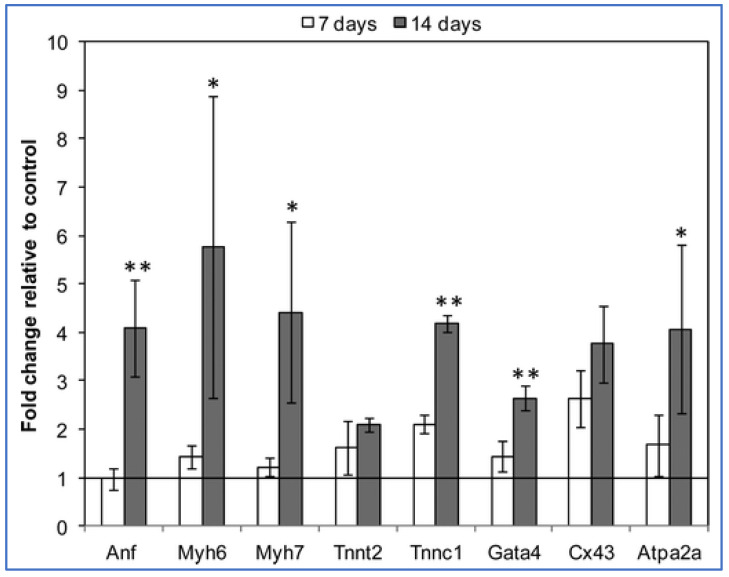
Gene expression data of cardiomyocytes cultured on chitosan/carbon scaffolds for 7 days and 14 days vs. control samples (chitosan scaffolds cultured using the same conditions) [33]. The fold change in gene expression is relative to the control (chitosan/carbon/cell constructs vs. chitosan/cell constructs). * *p* < 0.01; ** *p* < 0.05. (Anf = atrial natriuretic factor, Myh6/Myh7 = myosin heavy chain, Tnnc1 = troponin C type 1, Cx43 = gap junction α-1 or connexin 43, Atpa2a2 = calcium transporting ATPase).

It is known that the cardiac tissue is an electroconductive tissue capable of transferring electrical signals, which has made the development of the conductive materials for cardiac regeneration crucial [32]. Carbon nanofibers have been used to fulfill the functionality of electrical conductivity and have been used as the reinforcing filler for the biological matrices to improve tissue engineering functions. Chitosan can be reinforced with carbon nanotubes to form various types of composites for cardiac tissue engineering. The idea is to use the biocompatibility and biodegradability of chitosan with the electrical properties of carbon nanofibers. Evaluation of the cardiac gene expression profiles for the cardiomyocytes cultured in chitosan and chitosan/carbon scaffolds showed that most of the genes were overexpressed, specifically Troponin C type 1 (Tnnc1) and gap junction α-1 or connexin 43 (C × 43) in the order of 2- and 2.6 fold respectively, as shown in Figure 5. Tnnc1 is important for the contractile function of the cardiac muscle, whereas the C × 43 is important for the conduction of the electrical signals [33].

**Figure 6:**
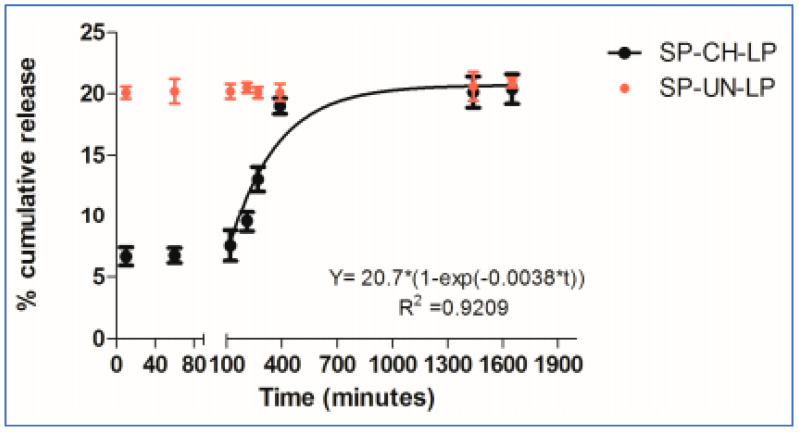
In vitro release of drug substance from chitosan-coated and uncoated liposomes in PBS (pH 7.4; 37 °C; *n* = 3) [35].

Gold nanoparticles (GNPs) were evenly dispersed throughout the chitosan (CS) matrix to provide electrical cues. The CS-GNP hydrogels were seeded with mesenchymal stem cells, and it has been shown that the CS-GNP scaffolds support the viability, metabolism, migration, and proliferation of MSCs [34]. Similarly, chitosan-coated liposomes are used to encapsulate the peptides and proteins that constitute the novel therapies for cardiac tissue regeneration [35]. Based on the evaluation data of the in vitro release of drug substance from chitosan-coated liposomes (CH-LP) and uncoated liposomes (UN-LP) performed in PBS at pH 7, 37 °C suggested a promising model for the sustained drug release, as shown in Figure 6 [35].

## 7. Chitosan-Based Cell Therapies for Coronary Heart Disease

In recent years, the administration of cells, especially cardiac progenitor cells or stem cells, is widely explored as a potential therapy for the regeneration of injured cardiac tissue as the result of MI. The most widely researched stem cells for cardiac applications include bone marrow stem cells (BMCs), mesenchymal stem cells (MSCs), embryonic stem cells (ESCs), hematopoietic stem cells (HSCs), induced pluripotent stem cells (iPSCs), and cardiac progenitor cells (CPC) [10,11,12,13]. Although widely explored, the success rate of these cell therapies is still under question. The reason for the same is the very low percent of cell viability in the harsh ischemic region as well as retention of these cells in the diseased region. To improve upon these constraints, polymeric biomaterials are used for the delivery of these cells. These materials could be fabricated into hydrogels, scaffolds, microcapsules, membranes, or more recently into 3D-printed structures [20,21]. The cells can be loaded into these structures and delivered into the ischemic region. The key advantage of these materials is that they can provide an extracellular matrix (ECM)-like microenvironment for the cells that helps in improved cell viability and retention. Moreover, these biomaterials can be tuned to release the required growth factors, immune-modulatory agents, and other factors required for cell survival as well as restoration of cardiac function [17,18,19,20,21]. These biomaterials can be classified as natural or synthetic based on the source from which they are obtained. Some of the natural polymeric biomaterials include chitosan, alginate, collagen, cellulose, hyaluronic acid, fibrin, silk, gelatin, and many more. Synthetic polymeric biomaterials are custom-made materials, with examples such as poly poly(lactic-co-glycolic acid) (PLGA), polycaprolactone (PCL), polylactic acid (PLA), PEG-based materials, poly(glycerol sebacate) (PGS), and many others [20,21].

Chitosan-based biomaterials, due to their versatility, biocompatibility, and biodegradability, are one of the most researched materials for drug delivery and tissue engineering applications. It was also found to have a better regenerative potential for cardiac tissue regeneration as well. Even in terms of cell delivery applications, chitosan has been very well researched. Chitosan-based hydrogels are most widely used for cell delivery applications. Other forms of chitosan that are used for cell delivery applications include microcapsules [36], coatings [37], nanofibrous scaffolds/patches [38], and recently 3D-printed structures [39]. Table 1 gives an exhaustive review of chitosan-based materials for cell therapy applications. In the case of cell delivery applications, chitosan is used alone or in combination with other polymers such as collagen, silk, dextran, alginate, PVA, and ECM to improve their mechanical and bioactive properties. There are also reports of chitosan in combination with electrical conduction-enhancing nanomaterials, especially gold nanoparticles and carbon nanomaterials, which are tabulated in Table 1.

## 8. Stem Cell-Based Therapies Utilizing Chitosan for Cardiac Disorders

**Figure 7:**
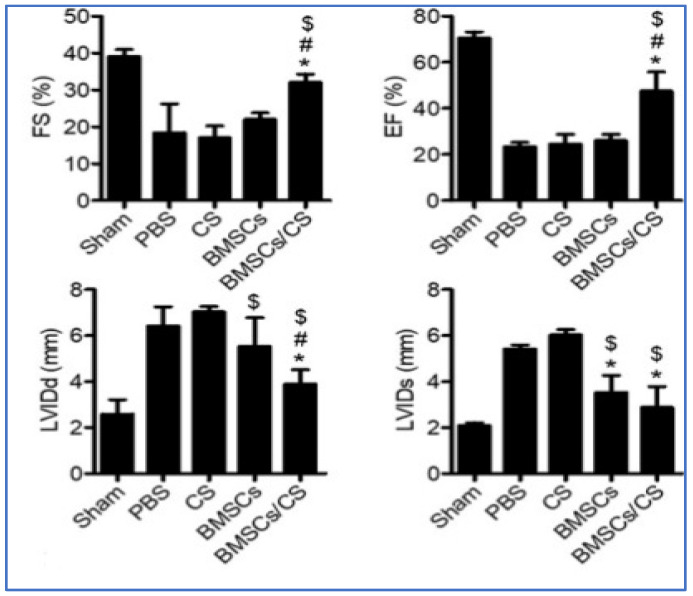
The levels of EF%, FS%, LVIDd, and LVIDd were evaluated using echocardiography (n = 12). The results suggested that co-transplantation of BMSCs with CS hydrogel significantly improved cardiac function. Data expressed as mean ± SEM. *n* = 8. * *p* < 0.05 versus BMSCs, # *p* < 0.05 versus BMSCs, $ *p* < 0.05 versus CS [81].

Stem cell-based therapies have the potential to fundamentally alter the conventional treatment of cardiovascular diseases (CVDs) by stimulating the regeneration of injured myocardium [80]. Several stem cells are considered for cardiovascular regeneration. Studies have used the mesenchymal stem cells (MSCs), which are pluripotent, found in the bone marrow, easy to isolate, and capable of differentiating into multiple lineages [69]. These characteristics have made MSCs a popular choice for stem cell-based therapy to treat heart failure. Chitosan’s thermoresponsive hydrogels are used for the retention of MSCs, increase the graft size in the ischemic heart, promoting MSC differentiation into myocytes, enhancing the impact of MSCs on neovasculature formation, and enhancing the effect of MSCs on the improvement of the cardiac function. Similarly, the chitosan-based injectable scaffold has been shown to improve the retention of embryonic stem cells in post-MI rats. Studies have shown that the major limitations of low cell survival and engraftment restrictions could be overcome by the co-transplantation of chitosan thermosensitive hydrogel with bone-marrow-derived MSCs (BMSCs) in a mouse model of MI [81]. CS hydrogel enhanced the BMSCs survival and recovery of cardiac function by protecting the vascular endothelial cells. It was observed that the BMSCs inhibited the inflammatory response and reduced the pyroptosis of vascular endothelial cells. Repairing heart function is evaluated by measuring four factors i.e., left-ventricular end-diastolic diameter (LVIDd), left-ventricular end-systolic diameter (LVIDs), left ventricular ejection fraction (EF), and fractional shortening (FS). The data suggests that the cardiac function in the case of BMSCs co-transplanted with CS hydrogels could significantly decrease the LVIDd and LVIDs of the hearts after infarction when compared to the PBS, CS, and BMSCs groups, as shown in Figure 7. In addition, the comparison of the PBS, CS, and BMSCs groups suggested that the BMSCs co-transplanted with CS hydrogels maintained the LV contractile function, including increased FS and EF.

Studies have shown that the chitosan (CS)/dextran (D)/β-glycerophosphate (β-GP) loaded with human mesenchymal stem cells (hMSCs) enhanced cardiac healing in acute myocardial infarction [82]. Bioactive chitosan hydrogel has also been shown to be promising in the stem cell regeneration of cardiac function. Studies have shown that the immobilization of the C domain peptide of insulin-like growth factor-1 on chitosan (CS-IGF-1C) to form a bioactive hydrogel when incorporated with human placenta-derived mesenchymal stem cells (hP-MSCs) can boost their therapeutic effects by their improved proliferation [83]. Thermoresponsive hydrogels have also shown promising application in vascularization and tissue repair in case of cardiac disorders. A recent study reported that the injectable hydrogel developed with chitosan, gelatin, β-glycerophosphate, and Arg-Gly-Asp (RGD) peptide, which is a thermoresponsive hydrogel, provides an ideal growth microenvironment for MSCs, smooth muscle cells, and endothelial cells [84]. The cell-inductive scaffold that can support the cardia cell behavior was designed by the incorporation of the optimized concentrations of the calcium silicate into the chitosan electrospun nanofibers to construct cardiac patch scaffolds [53]. These scaffolds have helped in the stimulation and the expression of cardiac-specific genes and proliferation of the neonatal rat cardiomyocytes (NRCMs).

## 9. Future Perspectives

With the advent of additive manufacturing, the tissue regeneration field is gearing up toward developing cell-laden 3D-printed tissue and organ mimics. Recently, 3D bioprinting is maturing as a technology, wherein bio-inks (a combination of polymeric materials with the appropriate cells of interest) are printed layer-by-layer to obtain 3D tissue or organ mimics. The challenge is to get the most suitable bio-inks for specific applications. In addition to the generation requirements such as biocompatibility and biodegradability, other primary characteristics of the bio-inks include its close match with the tissue’s mechanical properties, appropriate rheological characteristics, good printability, good cell-adhering properties, mimicking closely with the extracellular matrix (ECM) of the tissue of interests. Chitosan satisfies almost all the primary characteristics of the bio-ink mentioned above. There are already a few studies started in this area, wherein chitosan is optimized with other polymers to create bio-inks. In one study, hydroxybutyl chitosan (HBC), a thermo-responsive polymer, was used as a bio-ink for 3D bioprinting of cardiac tissue for regenerative medicine and pharmaceutical applications. They fabricated rectangular-shaped HBC gel on which human-induced pluripotent stem cell-derived cardiomyocytes (hiPSC-CM) and normal human cardiac fibroblasts (NHCF) coated with extracellular matrix (ECM) nanofilms are deposited layer-by-layer in a highly oriented fashion. They also co-cultured human cardiac microvascular endothelial cells (HMVEC), which resulted in highly vascularized and oriented cardiac tissue mimics [39].

There is also a review on chitosan as a biomaterial for 3D bioprinting, although its application in the area of 3D bioprinting of cardiac tissue is not explored much [85]. We expect this area to flourish in the coming years. Microfluidics is another area that is creating a big impact on the tissue engineering field. Various 3D tissue models are being developed using microfluidics-based techniques. There is a nice review on chitosan as a material for lab-on-a-chip devices. The specific applications of chitosan for cardiac applications are limited and are expected to surge in the coming years. For one instance, the chitosan-based injectable hydrogel is used as a scaffold that mimics the cell niche in a perfusion multi-chamber microbioreactor. The whole system behaves as an engineered heart tissue (EHT), which consists of neonate mice cardiac progenitor cells. This microbioreactor could be used as an in vitro 3D tissue model for drug screening applications. A clinical study of a hemostasis pad prepared using chitosan showed that it was useful for after invasive percutaneous procedures with the arterial approach. Arterial access is the key step during the endovascular treatment of cardiovascular diseases. The studies are in phase 4, which is an interventional and randomized treatment having 315 participants sponsored by Seoul National University Bundang Hospital, South Korea [86]. Recent studies indicated that different types of polymers such as alginate, gelatin methacryloyl (GelMA), hyaluronic acid, and chitosan are also utilized as injectable hydrogels in stem cell cardiac tissue repair [87].

## 10. Conclusions

Cardiac tissue engineering aims at supporting, replacing, or repairing cardiac tissue to improve functionality. The major issue with the viability of the implanted cells is addressed by the application of the polysaccharides, of which chitosan plays a major role. Chitosan helps in providing mechanical support, avoids the spread of the pro-inflammatory agents, and encloses the bioactive materials helpful for the regeneration of the cardiac tissue. Chitosan’s characteristics such as the positive charge and the hydrophilicity enable the creation of a soft tissue microenvironment, especially when blended with the biomolecules. Chitosan-based scaffolds help in the mechanical strength for the proliferation and differentiation of stem cells. With the increased research and understanding of the biological mechanisms of cardiac tissue regeneration and mechanical properties of the tissues, the chitosan-based strategies for cardiac tissue regeneration can be implemented better and improved.

## Figures and Tables

**Table 1:** List of various drug delivery systems designed using chitosan along with composition and their significant findings and use in cardiac therapies. [↑ indicates increase effect and ↓ decrease effect, respectively].

S. No	Type	Hybrid/CS Only	Components	Cell Types Used/Studied	Study Conclusion	Ref.
1	Injectable gel	Hybrid	Gelatin, β-glycerphosphate and Arg-Gly-Asp (RGD) peptide; also has stromal cell-derived factor-1 (SDF-1) nanoparticles and vascular endothelial growth factor (VEGF) nanoparticles.	Nil	↑ Vascularization in chick embryo chorioallantoic membrane (CAM) Model. ↑ heart function when compared to control group in rat myocardial infarction (MI) model	[40]
2	Cardiac patch	Hybrid	Cardiac extracellular matrix–chitosan–gelatin (cECM-CG) composite scaffold.	CD34 + endothelial progenitor cells (EPCs)	↑ Cell survival and proliferation ↑ differentiation of EPC toward endothelial cells	[41]
3	Injectable gel	Hybrid	Chitosan/dextran/β-glycerophosphate injectable hydrogel	Umbilical cord mesenchymal stem cells (UCMSCs)	↑ Cell viability and a linear controllable cell release rate. ↑ differentiation toward cardiac lineage	[42]
4	Injectable gel	CS	Temperature-responsive chitosan hydrogel	Somatic cell nuclear transfer- and fertilization-derived mouse embryonic stem cells	Performed in vivo mouse infarction model. ↑ 24 h cell retention and 4-week graft size ↑ differentiation into cardiomyocytes in vivo ↑ heart function at 4 weeks after transplantation ↑ the arteriole to venule densities within the infarcted area	[43]
5	Injectable gel	Hybrid	C domain peptide of insulin-like growth factor-1 embedded on chitosan (CS-IGF-1C)	Human placenta-derived mesenchymal stem cells (hP-MSCs)	Protect neonatal mouse ventricular cardiomyocytes (NMVCs) against oxidative stress in in vitro co-culture studies In in vivo mouse MI model, ↑ angiogenesis, ↓ fibrosis, ↓ apoptosis/inflammation	[44]
6	Injectable gel	Hybrid	Gold nanoparticles	Mesenchymal stem cells (MSCs)	↑ viability, metabolism, migration, and proliferation of MSCs ↑ differentiation of MSCs toward cardiomyocytes	[45]
7	Injectable gel	CS	Comparison between two injectable hydrogels (alginate, chitosan/β-glycerophosphate (chitosan/β-GP)) and two epicardial patches (alginate, collagen)	Human mesenchymal stem cells (hMSCs)	In vivo rat MI model: 8- fold ↑ in cell retention with alginate hydrogel; 14-fold ↑ in cell retention with chitosan/β-GP hydrogel; 47-fold ↑ in cell retention with collagen patches; 59-fold ↑ in cell retention with alginate patches	[46]
8	Cardiac patch by layer-by-layer (LbL)	Hybrid	Chitosan/silk fibroin-modified cellulose nanofibrous patches	Adipose tissue-derived mesenchymal stem cells (AD-MSCs)	In vitro cell studies: ↑ cell viability In vivo rat MI model: ↓ LV remodeling; ↓ LV end-diastole volume; ↓ LV end-systole volume; ↑ LV ejection fraction; ↑ fractional shortening; ↓ fibrosis; ↓ apoptosis; ↑ neovascularization	[47]
9	Injectable gel as drug delivery system	Hybrid	Chitosan-gelatin based gel loaded with ferulic acid (FA)	Cisd2 ++/−− iPSC-CM	↑ Sustained release of FA ↑ cell viability Good biocompatibility by subcutaneous injection in rabbit as well intramyocardial injection in Cisd2-/-C57BL6 mice studies.	[48]
10	Injectable gel	Hybrid	Alginate–chitosan hydrogel	Nil	In vivo rat MI model: ↓ Scar thickness, ↓ infarct expansion, and ↓ scar fibrosis; ↑ angiogenesis; ↑ recruitment of endogenous repair at the infarct zone; ↑ endogenous cardiomyocytes proliferation	[49]
11	Patch	Hybrid	Chitosan–poly vinyl alcohol (PVA)—carbon nanotubes (CNT) nanofibers	Rat mesenchymal stem cells (MSCs)	↑ Differentiation of MSCs towards cardiomyocytes. ↑ expression of Nkx2.5, Troponin I, and β–MHC cardiac marker	[38]
12	Injectable gel	CS	Chitosan injectable gel	Adipose-derived mesenchymal stem cells (ADSCs)	↑ Restoration of ROS-induced impairment of ADSC–matrix adhesion ↑ expression of integrin β1, integrin αV, p-FAK, p-Src, p-Akt ↓ expression of caspase 3 In rat MI model: ↑ engraftment and survival of transplanted stem cells ↑ homing of endogenous stem cells	[50]
13	Injectable gel as drug delivery system	Hybrid	bFGF-loaded CS injectable gel	Mouse embryonic stem cells (mESCs)	In vivo rat MI model: ↑ left ventricular ejection fraction (LVEF) ↑ LV fractional shortening (LVFS) ↑ arteriole densities within the infarcted area; ↓ Infarct size and fibrotic area	[51]
14	Injectable gel	CS	Chitosan injectable gel	Mouse embryonic stem cells (mESCs)	In vivo rat MI model: ↑ 24 h cell retention and 4-week graft size; ↑ heart function, wall thickness, and microvessel densities	[52]
15	Patch	Hybrid	Solubilized cardiac extracellular matrix (ECM), alginate, and chitosan	Human mesenchymal stem cells (hMSCs)	↑ Cell proliferation ↑ expression of cardiac marker (cTnT)	[53]
16	Hydrogel—Engineered heart tissue (EHT)	Hybrid	Chitosan-enhanced extracellular-matrix (ECM) hydrogel	Human induced pluripotent stem cell-derived cardiomyocytes (hiPSC-CMs)	ECM-EHT model for in vitro drug testing and screening	[54]
17	Hydrogel	Hybrid	A collagen–chitosan hydrogel	1. Human circulating progenitor cells (CPCs). 2. HUVECs	↑ Vascular-like structures when compared to collagen-only hydrogel ↑ vascular endothelial cadherin Expression—greater maturation of endothelial cells. In vivo subcutaneous mouse model: ↑ vascular growth ↑ von Willebrand factor (vWF+) and CXCR4+ endothelial/angiogenic cells	[55]
18	Injectable gel	Hybrid	Decellularized porcine cardiac extracellular matrix (pcECM) cross-linked with genipin alone or engineered with different amounts of chitosan	hMSCs	↑ Viability of hMSCs no immunogenicity ↑ cardiac function eight weeks post treatment	[56]
19	Patch	Hybrid	Decellularized myocardium extracellular matrix (ECM) and chitosan (CS)	Cardiac progenitor cells (CPCs)	In vitro cell studies ↑ cell viability and proliferation	[57]
20	Scaffolds/tissue engineered heart valves	Hybrid	Collagen–chitosan composite materials	Smooth muscle cells, fibroblasts, bone marrow mesenchymal stem cells (BMSCs)	↑ In vitro differentiation of BMSCs to endothelial cells ↑ 6-ketone prostaglandin content Stained positive for both smooth muscle actin and endothelial cell factor VIII	[58]
21	Injectable gel	Hybrid-electroconductive	Dextran-graft-aniline tetramer-graft-4-formylbenzoic acid and N-carboxyethyl chitosan	C2C12 myoblasts and human umbilical vein endothelial cells (HUVECs)	↑ Electroactivity and conductivity in the order of 10^−2^ mS/cm. C2C12 cells were released from the hydrogel matrix in a linear-like profile; ↑ cell proliferation ↑ regeneration of the skeletal muscle in a volumetric muscle loss injury model	[59]
22	Scaffold	Hybrid	Decellularized bovine pericardium extracellular matrix (DBPECM) coated with a layer of polycaprolactone–chitosan (PCL-CH) nanofibers	L-929, EA.hy926 cells and human umbilical cord mesenchymal stem cells (hUCMSC)	↑ Fibroblast and endothelial cell proliferation ↑ bio and hemocompatibility	[60]
23	Membrane	CS	CS membrane	Rat adipose-derived adult stem cells (ASCs)	Cells grown on this membrane forms spheroid. 20-fold ↑ expression of cardiac marker gene expression (Gata4, Nkx2-5, Myh6, and Tnnt2) when compared to the tissue culture polystyrene (TCPS) dish. in vivo MI model ↑ cardiac function increases	[61]
24	Hydrogel	CS	Chitosan thermosensitive gel	Bone marrow-derived mesenchymal stem cells (BMSCs)	↑ Cell survival ↓ inflammatory response ↓ pyroptosis of vascular endothelial cells ↑ heart function	[62]
25	Scaffolds	Hybrid	CS scaffold + carbon fibers	Neonatal rat heart cells	Has elastic modulus of 28.1 ± 3.3 KPa, similar to that measured for rat myocardium. Excellent electrical properties with a conductivity of 0.25 ± 0.09 S/m. ↑ expression of cardiac specific genes	[63]
26	Film	Hybrid	Chitosan–phosphorylcholine (CH-PC)	Bone marrow-derived cells (BMDC)	↑ Adhesion and proliferation of BMDC ↑ Endothelial differentiation ↑ EPC survival	[64]
27	Microcapsules	Hybrid	Fluorogenic genipin-cross-linked alginate chitosan (GCAC) microcapsules	Human adipose stem cells (hASCs)	↑ Expression of vascular endothelial growth factor (VEGF). Rat MI model: ↓ infarct size ↑ vasculogenesis, ↑ cardiac function; ↓ fibrosis	[36]
28	Linker molecule	Hybrid	Carboxymethyl chitosan as a linker molecule for PDA surfaces to attached vitronectin peptides	Human pluripotent stem cells (hPSCs)	↑ Reprogramming of human somatic cells into hiPSCs under defined conditions. ↑ proliferation and pluripotency of hPSCs. ↑ differentiation toward cardiomyocytes and neural cells	[65]
29	Scaffold	Hybrid	Chitosan–alginate scaffold	MSCs were obtained from the BM of Lewis male rats	In vitro cell studies: 40/60 alginate/chitosan PEC scaffolds—good mechanical and biological properties. In vivo rat MI model: ↑ cardiac function	[66]
30	Scaffold	Hybrid	Polyethylene glycol (PEG), hyaluronic acid, and chitosan	Human Wharton jelly mesenchymal stem cells (HWJMSCs)	In vivo rabbit MI model: ↑ cardiac function ↑ differentiation toward cardiomyocytes. ↑ neoangiogenesis	[67]
31	Hydrogel	Hybrid	Ti_3_C_2_ MQDs are incorporated into a chitosan-based hydrogel	Rat bone-marrow-derived mesenchymal stem cells; human iPSC-derived fibroblasts	↓ Activation of human CD4+IFN-γ + T-lymphocytes ↑ expansion of immunosuppressive CD4+CD25+FoxP3+ regulatory T-cells. ↑ conductivity ↑ cell survival and proliferation	[68]
32	3D-printed structure	Hybrid	Hydroxybutyl chitosan (HBC), with LbL assembly of gelatin and fibronectin	Human-induced pluripotent stem cell-derived cardiomyocytes (hiPSC-CM) with normal human cardiac fibroblasts (NHCF) and human cardiac microvascular endothelial cells (HMVEC)	Native organ-like three-dimensional (3D) cardiac tissue. ↑ alignment of hiPSC-CM and NHCF ↑ vascular network in orientation-controlled 3D cardiac tissue	[39]
33	Hydrogel	Hybrid	Graphene oxide quantum dot + chitosan + collagen hydrogel	Human mesenchymal stem cells (hMSCs)	↑ Angiogenesis; ↓ cardiomyocyte necrosis; ↑ cell survival factors; ↑ pro-inflammatory factors; ↑ pro-angiogenic factors and early cardiogenic markers. ↑ ejection fraction; ↓ fibrosis; ↓ infarct size	[69]
34	Patch	Hybrid	Calcium silicate (CS) was incorporated into the controllable aligned chitosan electrospun nanofibers	Neonatal rat cardiomyocytes (NRCMs)	↑ Cardiac and angiogenic specific markers; ↑ myofilament structure; ↑ aligned cell morphology; ↑ cell survival; ↑ Ca^2+^ transients of NRCMs. In vivo rat MI model: ↑ cardiac function; ↑ angiogenesis; ↓ scar size	[70]
35	Injectable gel	CS	Chitosan hydrogel	Rat bone marrow mesenchymal stem cells (MSCs)	↑ Graft size; ↑ cell retention in the ischemic heart, ↑ differentiation of MSCs toward cardiomyocytes; ↑ neo-vasculature formation; ↑ cardiac function and hemodynamics	[71]
36	Cardiac patch	Hybrid	Silk fibroin + CS + hyaluronic acid patch	Rat bone marrow MSCs	↑ Cell viability and proliferation; ↑ expression of Gata4, Nkx2.5, Tnnt2, and Actc1 genes; ↑ expression of cardiotin and connexin 43.	[72]
37	Membrane	CS	CS membranes	Adipose-derived adult stem cells (ADAS)	Spheroid formation and cardiomyogenic differentiation of MSCs on chitosan membranes	[73]
38	Microcapsules	Hybrid	Alginate–chitosan–alginate shell on a liquid core containing ES cells	Encapsulation and culture of embryonic stem (ES) cells in the liquid core of microcapsules	↑ Cell survival and proliferation 8.2-fold ↓ immunoglobulin G (IgG) binding to the cells.	[74]
39	Coating on metallic stents	Hybrid	Metallic stents are coated with CS–hyaluronic acid–antibody	CD133 stent for HSC capture	CD133 stent—selectively capture hematopoietic stem cells (HSC), which directionally differentiate into vascular ECs	[37]
40	Hydrogel	CS	Chitosan hydrogel	Brown adipose-derived stem cells (BADSCs)	↑ Cardiac differentiation of BADSCs; ↑ survival of BADSCs; ↑ angiogenesis; ↑ heart function; ↓ adverse matrix remodeling	[62]
41	Patch	Hybrid	CS–Collagen scaffold. Negative replica patterning based on electrophoretic deposition to realize multi-scale micro-structured Chitosan–collagen (C/C) scaffolds	Rat neonatal cardiomyocytes (rCM)	↑ Attachment, spreading, and orientation of human CMs	[75]
42	Patch	Hybrid	Chitosan–hyaluronan/silk fibroin patches	Nil	↓ Dilation of the inner diameter of left ventricle (LV); ↑ wall thickness of LV; ↑ neovascularization; ↑ secretion of VEGF	[76]
43	Hydrogels	Hybrid	Collagen–chitosan composite hydrogels-controlled release of thymosin β4	Nil	Controlled release of thymosin β4 for 28 days; ↑ cell migration ↑ neovascularization	[77]
44	Hydrogel	Hybrid	Peptide-modified chitosan–collagen hydrogel	Cardiomyocytes (CM)	↑ Retention of CMs	[78]
45	Hydrogel	Hybrid	RoY peptide conjugated CS chloride thermogel	Human umbilical vein endothelial cells (HUVEC)	↑ Survival, proliferation, migration of HUVEC; ↑ tube formation; ↑ angiogenesis and ↑ cardiac function in rat MI model.	[31]
46	Hydrogel	CS	Chitosan-based pH-responsive hydrogel	human Bone Marrow Mesenchymal Stem Cells (hBMSCs) and human Adipose Mesenchymal Stem Cells (hADSCs)	↑ Cell survival and proliferation	[79]

## Data Availability

Not applicable.
